# Wavefront Line‐Scan Imaging Via a Single‐Pixel Detector

**DOI:** 10.1002/advs.75485

**Published:** 2026-04-30

**Authors:** Nuo Liu, Aiping Zhai, Tingting Zheng, Wenjing Zhao, Dong Wang

**Affiliations:** ^1^ College of Physics and Optoelectronics Engineering Taiyuan University of Technology Yingze P. R China; ^2^ Shanxi Key Laboratory of Precision Measurement Physics Taiyuan University of Technology Yingze P. R China; ^3^ Key Laboratory of Advanced Transducers and Intelligent Control System Ministry of Education and Shanxi Province Taiyuan University of Technology Yingze P. R China

**Keywords:** 1‐D spatial light modulation, single‐pixel detection, wavefront imaging

## Abstract

Wavefront single‐pixel imaging (WSPI) has emerged as a promising approach for simultaneous amplitude and phase reconstruction, especially in spectral wavebands where cameras are immature or even unavailable. However, conventional WSPIs suffer from limited imaging speed & range, and heavy pattern‐data storage. Here, we demonstrate a wavefront line‐scan imaging (WLSI) technique that integrates 1D spatial light modulation with single‐pixel detection, enabling faster imaging in a large rectangular range with minimal pattern‐data storage. A theoretical model is developed and validated by simulations and experiments. A digital micromirror device (DMD) worked in 1D, achieving a modulation rate of 90.9 kHz, enabling line‐scan imaging at 2.8 ms per line without compressive down‐sampling. Consequently, the WLSI achieves over 4‐fold enhancement in imaging speed and significantly reduces the pattern‐data storage to more than 5 orders of magnitude as compared to the WSPI, when reconstructing a wavefront of 256 × 256 pixels at full sampling. The push‐broom WLSI extends the imaging range, enabling continuous acquisition in a long rectangular range. Combining the advantages of WSPI with the flexibility of line‐scan imaging, we offer a potential solution for high‐throughput amplitude and phase imaging of biological samples, defect detection of optical components, wavefront detection in special spectra, etc.

## Graphical abstract

We propose and demonstrate a wavefront line‐scan imaging paradigm that integrates 1D spatial light modulation with single‐pixel detection. Benefiting from 1D modulation, not only is the imaging speed improved, but also the storage of modulation patterns is reduced by several orders of magnitude. It thus enables faster high‐throughput amplitude and phase imaging with minimal pattern‐data storage.

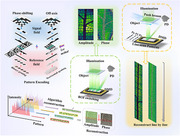

## 1 Introduction

Wavefront imaging technology [1, 2, 3, 4] reproduces the amplitude and phase of the light wave, with extensive applications in optical system calibration [5], transparent sample observation [6], etc. [7, 8, 9]. Current wavefront sensing, which relies on pixelated array sensors (e.g., CCD/CMOS), poses a challenge for special‐wavelength detection, like mid‐infrared [10] and ultraviolet, where sensors are either immature [11] or costly to manufacture [12]. Subsequently, point detection [13, 14, 15] with optical interferometry [16] has been developed as one of the effective solutions, known as wavefront single‐pixel imaging technology (WSPI) [17, 18, 19, 20, 21] in recent years. It employs only a single‐pixel detector and the time‐varying modulation optical field to recover the wavefront computationally. Benefited from these simple and available sensors, WSPI enables low‐light and special‐band wavefront detection that struggles to do with array sensors [22], which have been applied in biomedical imaging [23, 24], terahertz imaging [25, 26, 27], digital holography [28, 29], and microscopy [30, 31].

As a sacrifice, the imaging speed in WSPI remains a major obstacle since a large number of modulations are necessary. Researchers have focused on resolving it via several methods. One approach is to explore the common‐path off‐axis WSPI [32] instead of the phase‐shifting one [33, 34, 35] by avoiding multi‐step phase encoding, improving the speed by several times [36, 37]. Alternatively, compressed sensing (CS) algorithms [38, 39, 40, 41] can be adopted to accelerate by reducing the number of measurements. However, the former still has limitations in speed, while the latter suffers from reconstruction quality degradation when the target structure is complex and lacks sparsity in the frequency domain. Thus, there remain challenges to strike a balance between imaging quality and speed.

In terms of modulators, digital micromirror devices (DMDs) behave feebly in pursuing higher speed WSPI despite their modulation rates being around 22.7 kHz [42, 43, 44]. It has been reported that DMD‐based WSPI with the off‐axis configuration reaches 1.3 frames per second (fps) at a resolution of 128 × 128 pixels [45]. In addition, the limited on‐board memory volume of DMD hinders the development of high‐resolution WSPI. Specifically, the higher the resolution it requires, the larger the number of patterns that need to be stored in DMD, leading to memory overflow unless using down‐sampling strategies.

Here, we propose to do wavefront line‐scan imaging (WLSI) using 1D spatial light modulation with single‐pixel detection rather than the 2D counterpart. By switching the line‐scan patterns (LPs) within a progressive scanning over the target, the amplitude and phase of the wavefront can be simultaneously captured using a DMD working in 1D spatial light modulation and a photodetector (PD). Compared with the 2D modulation‐based WSPI, WLSI achieves line‐scan imaging at 2.8 ms per line without compressive down‐sampling, due to the slender, higher speed updating (90.9 kHz) of the LPs. Meanwhile, WLSI allows high‐resolution wavefront imaging with extremely small pattern‐data storage demand. The concept can be demonstrated using two different scanning modes, in which push‐broom scanning can theoretically realize an infinite imaging range along the scanning direction. The proposed method can achieve high‐throughput amplitude and phase imaging of biological samples.

## 2 Principle

### 2.1 Pattern Encoding

By extracting row vectors from a conventional 2D modulation basis, the 1D basis, i.e. *P_n_* (*x*) (*n* ∈ (1, *N*)), is obtainable, such as the Hadamard basis, the Fourier basis, and the discrete cosine transform (DCT) basis. Mathematically, these 1D bases are orthogonal to each other. The Hadamard basis is employed as the sensing matrix here because it is a binary matrix with elements of only 1 and ‐1, making it more suitable for a binary‐modulation device such as a DMD. Other orthogonal bases and non‐orthogonal bases can be substituted as well, and more details can be seen in Section S3.

**FIGURE 1:**
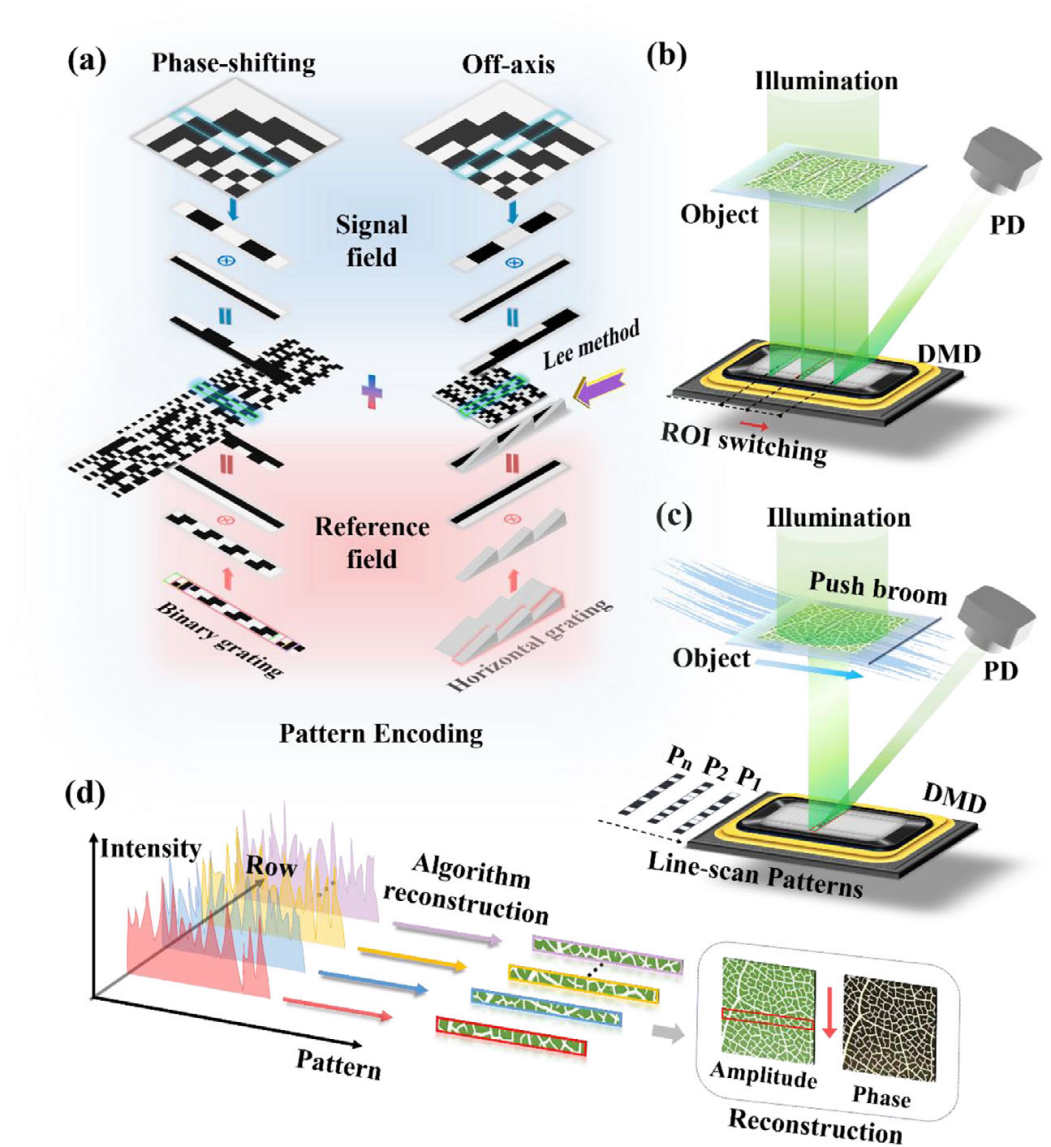
Schematic of the WLSI. (a) Schematic illustration of line‐scan patterns generation based on phase‐shifting interferometry and off‐axis interferometry. (b) The ROI switching strategy. (c) The push‐broom strategy. (d) Reconstruction process of the WLSI.

As shown in Figure 1a, based on phase‐shifting interferometry and off‐axis interferometry, we have established a WLSI framework, respectively. This framework employs the reference strategy to divide an unknown field into the signal and reference parts. To control the relative phase between the reference and signal fields, the signal part remains unchanged, while the reference part is specifically modulated.

Specifically, for phase‐shifting WLSI: A binary grating is introduced into the reference part to achieve four‐step phase shifts [44]. The signal and reference fields are then superimposed, which produces the phase‐shifting encoded LPs. The corresponding mathematical derivation is provided in Section S1. For off‐axis WLSI: A preset oblique grating (its angle is around 0.66°) is added into the reference component to introduce the phase difference required for off‐axis interference. Then, the signal and reference fields are superimposed and subsequently converted into a binary pattern to control the DMD, for achieving the desired modulation of the target wavefront. The mathematical derivation for this process is given in Section S2.

A detailed comparison of using different methods encoding the DMD to achieve the necessary modulation for the above‐mentioned phase‐shifting interference and off‐axis interference can be found in Section S5. Subsequently, phase‐shifting WLSI or off‐axis WLSI can be realized using two scanning methods, i.e., the Region of Interest (ROI) switching strategy (RS‐WLSI) in Figure 1b and the push‐broom strategy (PB‐WLSI) in Figure 1c.

### 2.2 WLSI Realized Using the ROI Switching Strategy (RS‐WLSI)

In the RS‐WLSI shown in Figure 1b, the object is illuminated by a Gaussian beam, and the light carrying the object's information is relayed to the DMD surface through a 4‐f optical system. A narrow rectangular ROI is defined on the DMD, which modulates a single line of the object. Preloaded LPs are rapidly updated at a refresh rate of 90.9 kHz. The modulated light is collected by a lens and synchronously recorded by a PD. Then, a one‐line image of the object can be obtained by correlating the recorded intensity values and the LPs. Subsequently, by shifting the ROI on the DMD and repeating the line‐scan imaging, one can reconstruct a 2D image of the object.

### 2.3 WLSI Realized Using the Push‐Broom Strategy (PB‐WLSI)

WLSI can also achieve wavefront sampling by pushing‐broom across the object according to the principle shown in Figure 1c. Here, the ROI is fixed at the center of the DMD. The object placed on an electric displacement stage is illuminated by a Gaussian beam, and the transmitted light is relayed onto the DMD surface. Preloaded LPs are rapidly updating. The modulated light is collected by a lens and synchronously recorded by a PD. Then, a one‐line image of the object can be reconstructed. Subsequently, the object translates along the vertical direction of the ROI at a speed higher than the DMD refresh rate, ensuring the object remains relatively stationary. Through the synchronized coordination of “pattern scrolling and object movement”, PB‐WLSI can be realized.

As illustrated in Figure 1d, whether it is the RS‐WLSI or PB‐WLSI, by correlating the recorded intensity values and the LPs, a line of an object can be reconstructed by the second‐order correlation (SOC) algorithm. Repeating this process line‐by‐line, a 2D wavefront information of the object can be obtained.

## 3 Experimental Results

### 3.1 Experimental Verification of RS‐WLSI

RS‐WLSI is demonstrated by retrieving the amplitude of a USAF target and the phase of a Siemens star. The details of the experimental setup can be found in Section S7. Both phase‐shifting RS‐WLSI and off‐axis RS‐WLSI share the same setup. The narrow rectangular ROI is set as 128 imaging pixels, and each imaging pixel consists of 4 × 4 DMD micromirrors. The peripheral reference strategy (up and down) was employed here, and other reference strategies, such as checkerboard and self‐reference strategies, are also simulated and demonstrated experimentally in Section S4.

**FIGURE 2:**
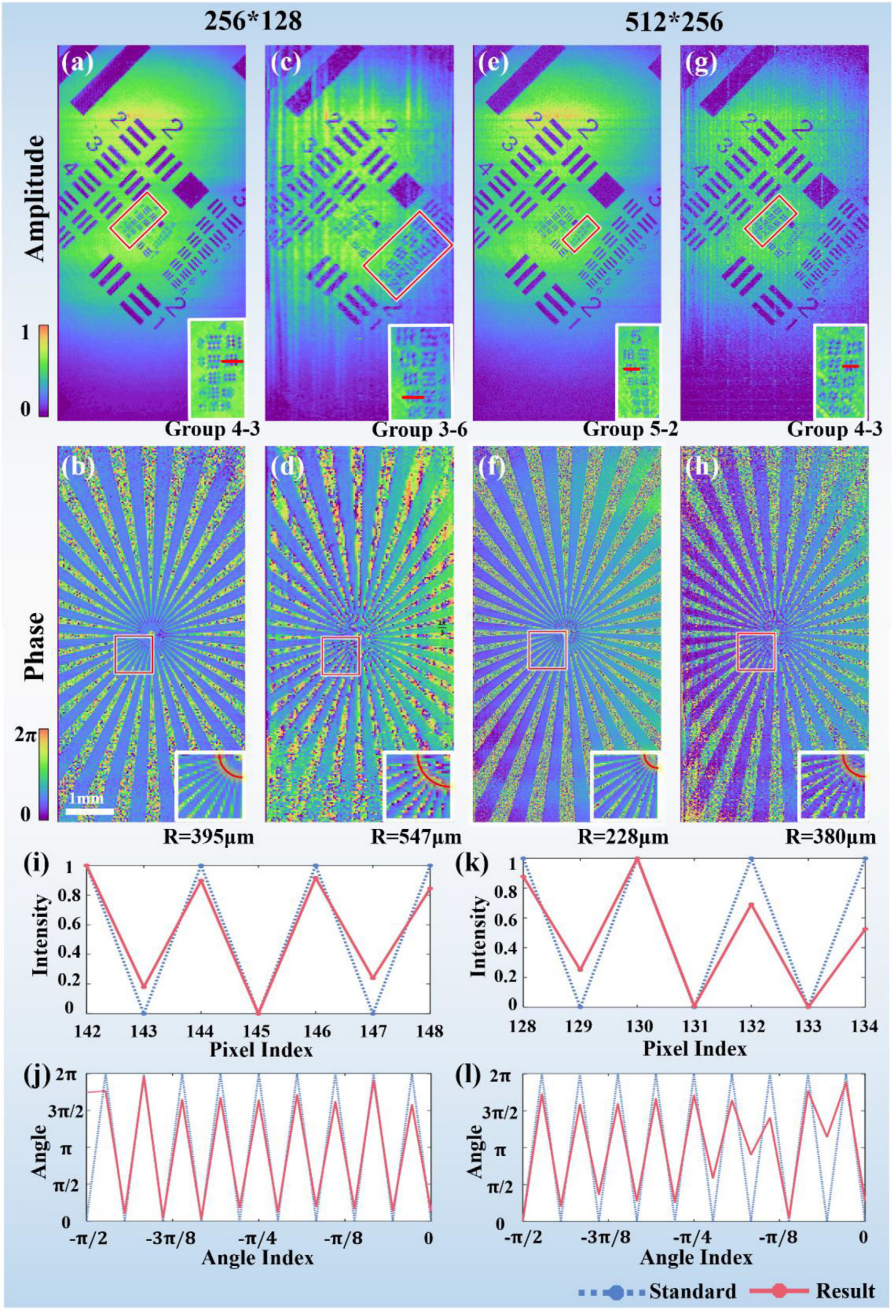
Reconstructed amplitude and phase of the standard test targets using the phase‐shifting RS‐WLSI and off‐axis RS‐WLSI. (a–d) These are the reconstructed results after conducting 256 times line‐scan imaging, respectively. (e–h) are the reconstructed results after conducting 512 times line‐scan imaging, respectively. (i–l) indicate the experimental data along the red lines marked in (e)‐(h), respectively.

The imaging results of phase‐shifting RS‐WLSI and off‐axis RS‐WLSI are shown in Figure 2a–d. To facilitate visualization, the red regions in the reconstruction results are zoomed in. The finest identifiable element is the third one in the fourth group (abbreviated as 4‐3, with 24.80 µm linewidth) in the phase‐shifting method, whereas the off‐axis method corresponds to 3–6 (35.08 µm linewidth), which is consistent with the physical resolution of the system (30.4 µm). Additionally, the phase information at a specified radius *R* (from −π/2 to 0) within the Siemens star image was evaluated, and more details are provided in Section S8. The minimum radius for the analyzable phase petals was *R* = 395 µm for the phase‐shifting and *R* = 547 µm for the off‐axis reconstruction. The results confirm the effectiveness of the proposed method.

Interestingly, one can adjust the 4 × 4 merged DMD micromirrors to be 2 × 2 merged ones to enhance the imaging resolution, as shown in Figure 2e–h, while maintaining the same field of view (FOV) size as results in Figure 2a–d. For the amplitude, the finest identifiable element is refined to 5‐2 (13.92 µm linewidth) with the phase‐shifting method and 4‐3 (24.80 µm linewidth) with the off‐axis method. Furthermore, a quantitative comparison between the experimental cross‐section distribution and theoretical values is presented in Figure 2i,k, where the experimental data align with the theoretical line, both exhibiting three distinct depressions. For phase reconstruction, the radius of expansion of the phase petals toward the center markedly decreased: from 395 to 228 µm for phase‐shifting WLSI, and from 547 to 380 µm for off‐axis WLSI (Figure 2j,l). The results demonstrate that the reconstructed quality aligns with the standard petal morphology. Although the off‐axis RS‐WLSI exhibits slightly inferior imaging quality compared to phase‐shifting RS‐WLSI, it demonstrates faster amplitude and phase recovery.

The experimental results validate the feasibility of the RS‐WLSI. Due to the setting narrow ROI, the maximum refresh rate of DMD can reach 90.9 kHz. Therefore, the off‐axis RS‐WLSI can achieve line‐scan imaging of 2.8 ms per line at a horizontal resolution of 256 pixels with full sampling.

### 3.2 Experimental Verification of PB‐WLSI

**FIGURE 3:**
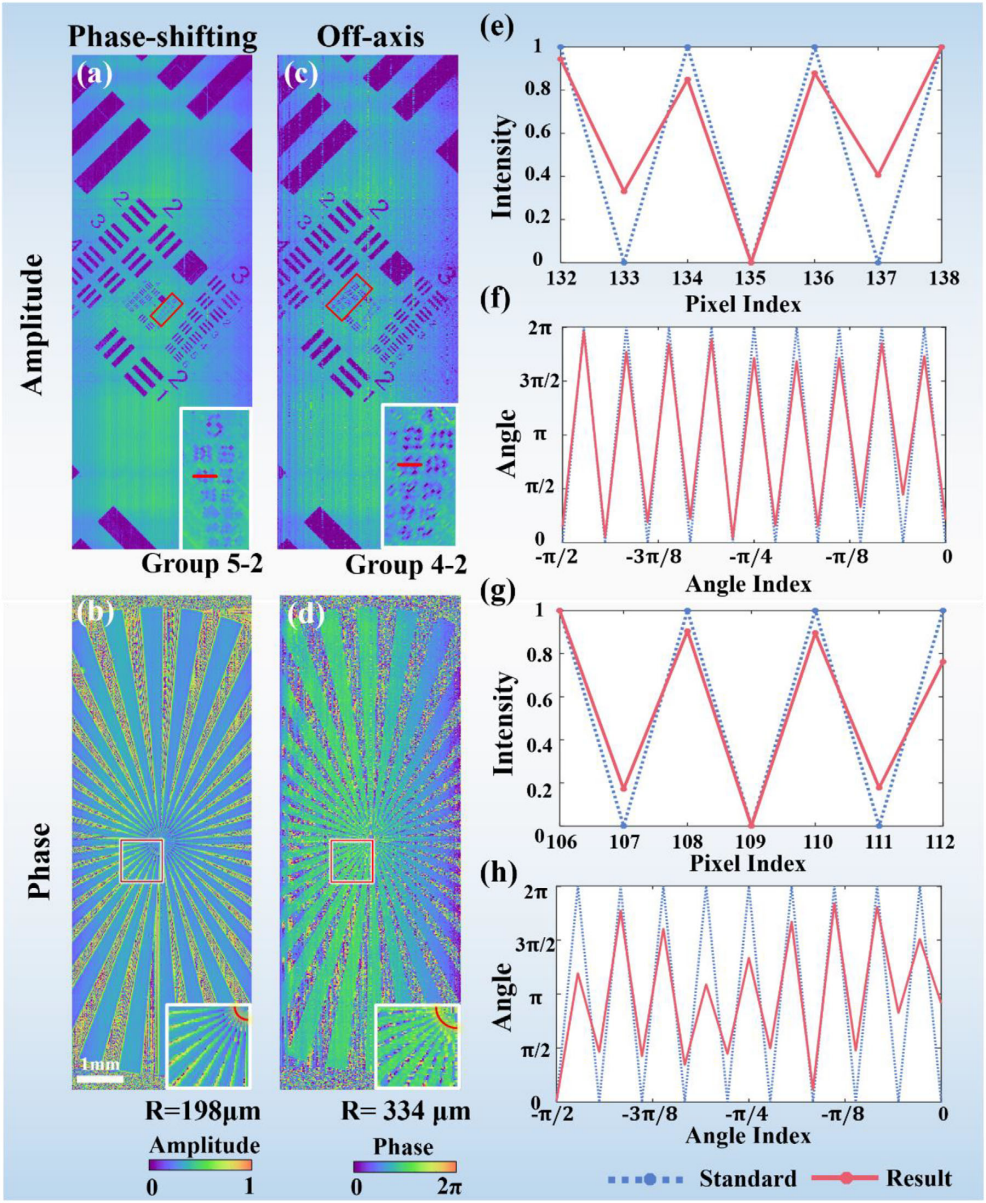
Reconstructed amplitude and phase of the standard test targets after conducting 700 times line‐scan imaging. (a, b) These are the reconstructed results of phase‐shifting WLSI, respectively. (c, d) These are the reconstructed results of off‐axis WLSI, respectively. (e–h) indicate the experimental data along the red lines marked in (a)‐(d), respectively.

For PB‐WLSI, the same targets are used for concept validation. The narrow rectangular ROI is set as 256 imaging pixels, and each imaging pixel consists of 2 × 2 DMD micromirrors. The reconstruction results obtained with phase‐shifting PB‐WLSI and off‐axis PB‐WLSI are shown in Figure 3. Intuitively, for the amplitude reconstruction results in Figure 3a,c, element 5‐2 (13.92 µm linewidth) is clearly identified using the phase‐shifting method, and the off‐axis counterpart is 4‐2 (27.84 µm linewidth). For the phase reconstruction results in Figure 3b,d, the minimum analyzable radii for the nine pairs of phase petals are *R* = 198 µm and *R* = 334 µm, respectively. The corresponding 1D profiles are provided in Figure 3e‐h, which are consistent with the standard one.

Compared with the RS‐WLSI, the PB‐WLSI can theoretically realize an infinite imaging range along the scanning direction. However, its imaging speed is limited by the translational speed of the displacement stage. Luckily, there are displacement stages that can match the refresh rate 90.9 kHz [46].

### 3.3 WLSI Compared to the State‐of‐the‐Art WSPI

Previous experiments have validated the feasibility of the RS‐WLSI and PB‐WLSI. To further demonstrate their performance, a comparison with the state‐of‐the‐art WSPI is performed. In the state‐of‐the‐art off‐axis WSPI [36], a total of 65536 modulation patterns is required to reconstruct an image with a resolution of 256 × 256 pixels at full sampling. However, the storage capacity of the DMD (Vialux V‐6501, 128 Gbit onboard memory) is limited to 62137 binary patterns, which means that the DMD cannot store all patterns at once. For phase‐shifting WSPI, this issue is more prominent because it requires a larger storage capacity. In contrast, the memory occupation of the off‐axis WLSI proposed is only 0.94 Mbit, achieving over 5 orders of magnitude reduction compared to the state‐of‐the‐art off‐axis WSPI.

**FIGURE 4:**
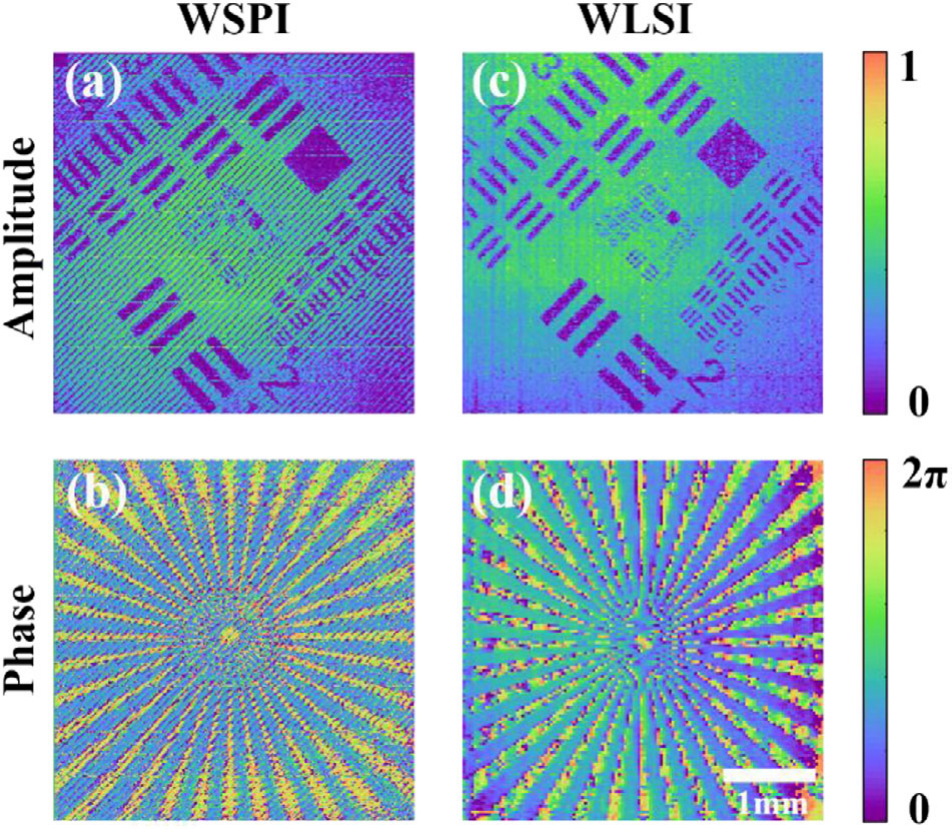
Comparison of imaging results of WLSI and WSPI. (a, b) These are respectively the reconstructed results of off‐axis WSPI. (c, d) These are respectively the reconstructed results of off‐axis WLSI.

The imaging results of the off‐axis WSPI and off‐axis WLSI are shown in Figure 4. It can be observed that the quality of off‐axis WLSI did not deteriorate. In addition, the WLSI can leverage the rolling and switching capabilities of the ROI to elevate the DMD refresh rate to 90.9 kHz, compared to WSPI's 22.7 kHz. Its imaging speed is thus enhanced more than 4‐fold.

### 3.4 High‐Throughput Amplitude and Phase Imaging of Biological Samples

**FIGURE 5:**
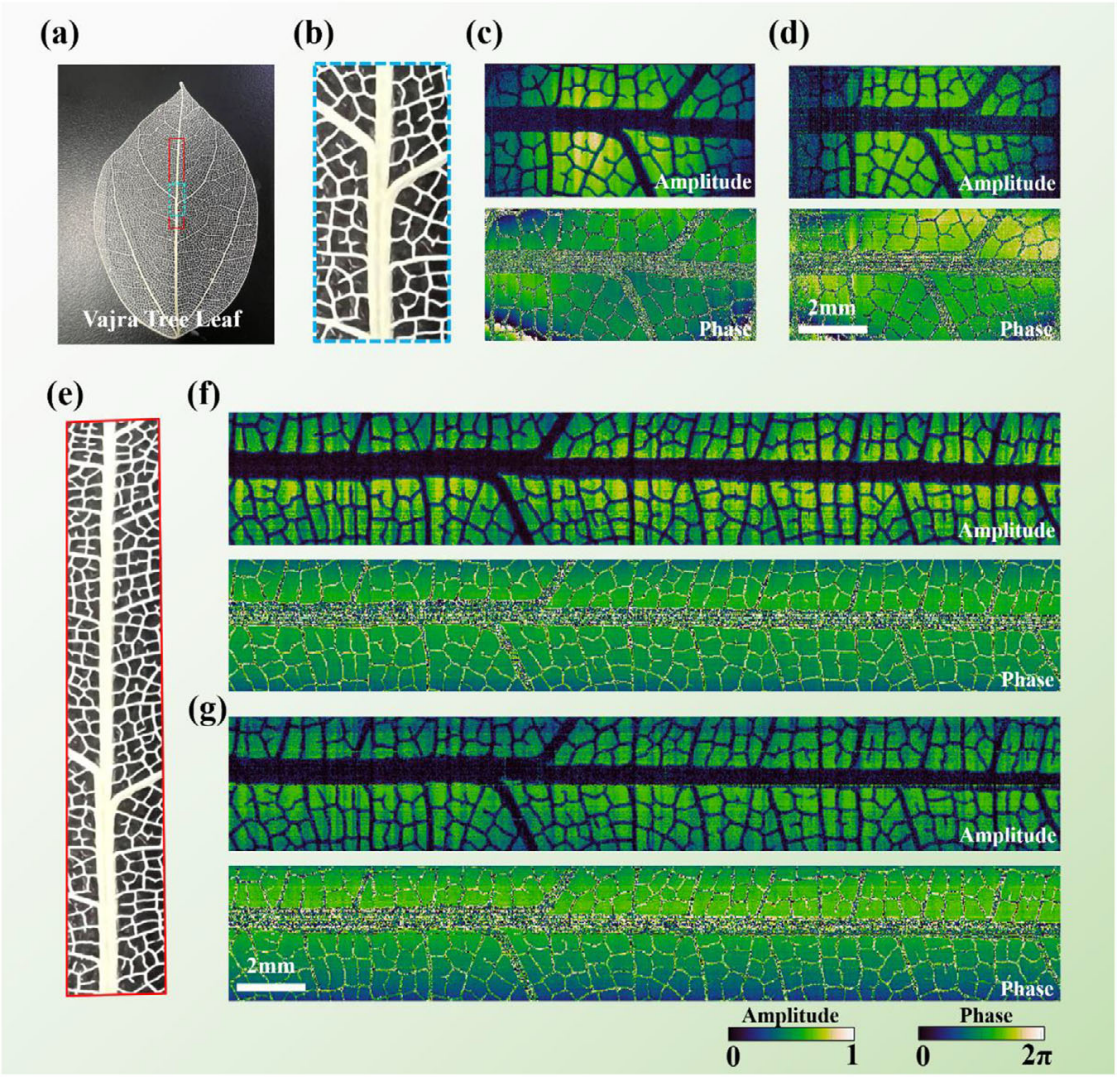
Reconstructed amplitude and phase of Vajra tree leaf. (a) Ground truth. (b) and (e) are respectively the zoom‐in of the blue and red rectangular areas in (a). (c, d) are respectively the reconstructed results of phase‐shifting RS‐WLSI and off‐axis RS‐WLSI with an imaging resolution of 512 × 256 pixels and an imaging range of 7.8 mm × 3.9 mm. (f, g) are respectively the reconstructed results of phase‐shifting PB‐WLSI and off‐axis PB‐WLSI with an imaging resolution of 1600 × 256 pixels and an imaging range of 24.3 mm × 3.9 mm.

To characterize the wavefront imaging capability of WLSI for biological samples, a Vajra tree leaf is selected as the target. Figure 5b,e are the zoom‐in ground truths of the red and blue areas in the sample. Figure 5c,d are the reconstructed results using phase‐shifting RS‐WLSI and off‐axis RS‐WLSI with an imaging resolution of 512 × 256 pixels and an imaging range of 7.8 mm × 3.9 mm. Figure 5f,g show the counterpart using PB‐WLSI, with an imaging resolution of 1600 × 256 pixels and an imaging range of 24.3 mm × 3.9 mm.

**FIGURE 6:**
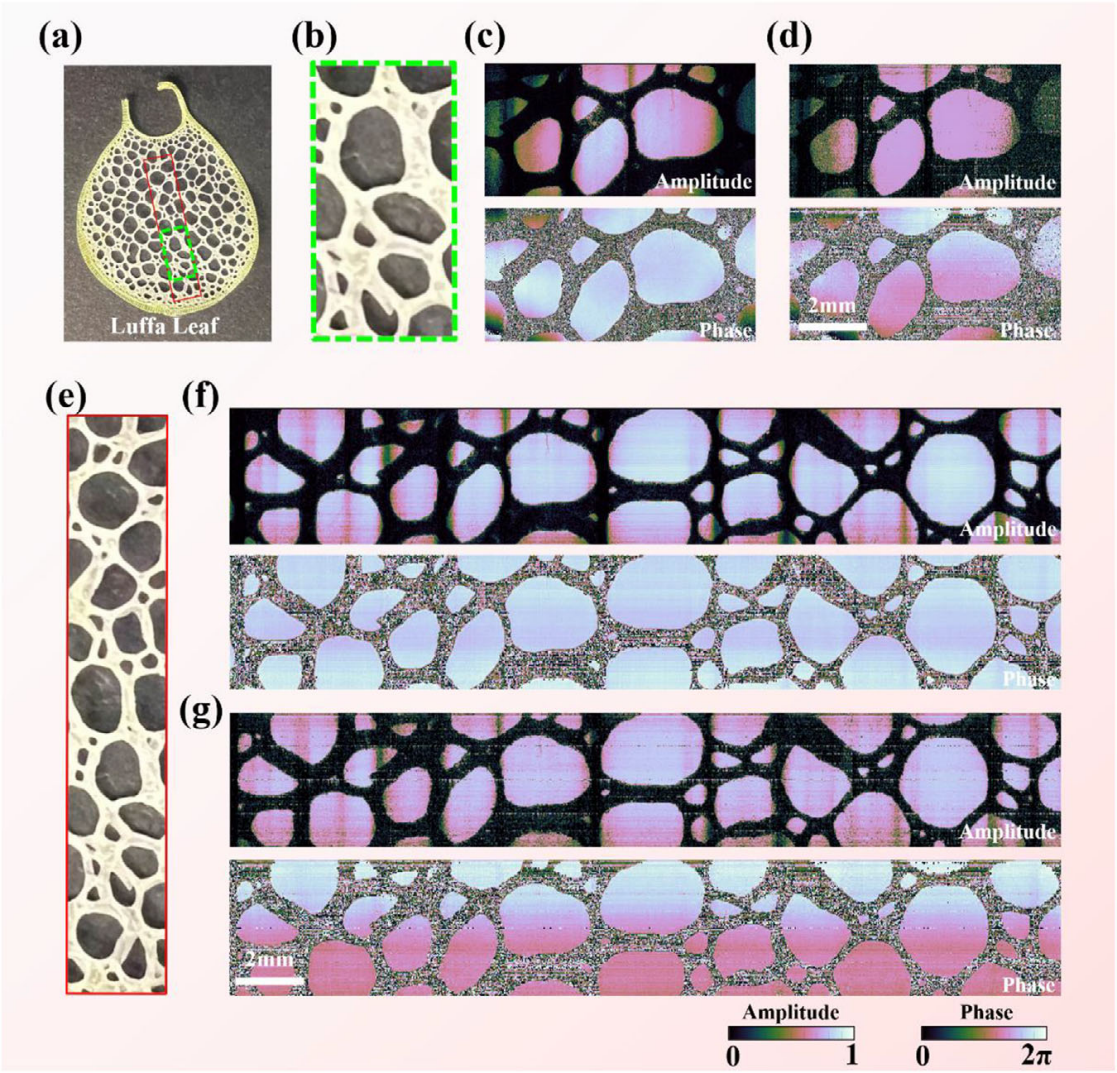
Reconstructed amplitude and phase of a leaf of Luffa slice. (a) Ground truth. (b, e) are respectively the zoom‐in of the green and red rectangular areas in (a). (c, d) are respectively the reconstructed results of phase‐shifting RS‐WLSI and off‐axis RS‐WLSI with an imaging resolution of 512 × 256 pixels and an imaging range of 7.8 mm × 3.9 mm. (f, g) are respectively the reconstructed results of phase‐shifting PB‐WLSI and off‐axis PB‐WLSI with an imaging resolution of 1600 × 256 pixels and an imaging range of 24.3 mm × 3.9 mm.

Specifically, the chemically treated Vajra tree leaf retained only its venation structure. The veins partially blocked light while the intervening tissue transmitted it, enabling clear structural identification, as evidenced by the results shown in Figure 5c,d,f,g. The leaf surface exhibits a texture with convex and concave features, which are clearly distinguished in the phase images and reflect changes in tissue thickness. These structural characteristics may play important roles in physiological functions such as water retention and photosynthesis. The whole imaging process for the Vajra tree leaf is shown in Video S1. Another biological sample, a Luffa leaf slice shown in Figure 6, reveals a distinct, irregular, circular, vein skeleton structure that contrasts with the network structure of the Vajra tree leaf. This further confirms the effectiveness of WLSI. The whole imaging process for the Luffa leaf slice is shown in Video S2.

The experimental results demonstrate that the proposed WLSI accurately recovers the wavefront of biological samples with a large area in both phase‐shifting and off‐axis configurations, confirming its good performance in biological detection. This technique offers distinct advantages for analyzing structural details by enabling continuous, non‐destructive observation under uniform conditions (e.g., of leaf venation and surface topography), thereby facilitating direct comparison of spatial heterogeneity and functional morphology within tissues.

## 4 Discussion and Conclusion

We have demonstrated a method employing 1D spatial light modulation with single‐pixel detection to enable faster wavefront imaging in a large rectangular range with minimal pattern‐data storage. By controlling the relative movement between the modulation area and the target, two scanning approaches, RS‐WLSI and PB‐WLSI, have been developed to implement the concept, almost sharing the same very simple yet powerful setup. The experimental results validate the effectiveness of this concept and show its advantages. The proposed method has been applied for amplitude and phase imaging of biological samples, achieving high‐throughput, clear reconstruction of leaf vein structure and surface morphology, demonstrating its potential for application in biological imaging.

Here, two scanning approaches, RS‐WLSI and PB‐WLSI, present their individual advantages for different imaging scenarios and can be selected flexibly according to different imaging demands. Specifically, RS‐WLSI performs scanning through ROI switching without moving the target, thereby avoiding mechanical perturbation and error. For PB‐WLSI, pushing‐broom the target enables imaging with an infinite imaging range along the scanning direction theoretically and overcoming edge dark field caused by fixed Gaussian illumination. They fully utilize the high refresh rate characteristics of DMD within ROIs, accelerating the imaging speed and reducing the pattern‐data storage amount.

Compared with the existing state‐of‐the‐art WSPI, the proposed WLSI accelerates imaging speed by more than 4 times and achieves a remarkable memory reduction of over 5 orders of magnitude, yet maintains comparable imaging quality when reconstructing a wavefront of 256 × 256 pixels at full sampling. Moreover, the proposed WLSI in push‐broom mode extends the imaging range, enabling continuous acquisition over a long rectangular range. Nevertheless, a minor stitching error is introduced during wavefront line‐scan reconstruction with our method, due to the subtle differences between adjacent lines. Interestingly, it is found that the higher the resolution of the reconstruction, the weaker the impact of this error, as verified by the comparison between Figures S8 and S9 in Section S9. Notably, the minor stitching error could be eliminated by referring to existing interferogram stitching methods, which can further improve the reconstruction accuracy.

The DMD worked in 1D spatial light modulation, can achieve a modulation rate of 90.9 kHz, enabling line‐scan imaging of 2.8 ms per line at a horizontal resolution of 256 pixels without compressive down‐sampling. The down‐sampling strategies can further improve imaging speed at the expense of imaging quality. At 25% sampling, the imaging speed of WLSI can be increased to 0.7 ms per line, as described in Section S6. Adaptive sampling schemes and deep learning‐optimized sampling strategies are expected to further enhance reconstruction quality at low sampling rates.

In terms of hardware architecture, the adoption of high‐speed precision electric translation stages can further improve stepping precision, while optimized control algorithms enable high‐speed synchronization with DMD modulation, which would significantly improve the scanning stability and imaging efficiency of PB‐WLSI. This work is demonstrated with a 1D spatial light modulation speed of 90.9 kHz by utilizing the ROI function of a DMD. Adopting an existing 1D phase modulator with a 350 kHz operating frequency [47] is expected to further enhance the imaging speed. Additionally, replacing the 1D modulator with a fast‐spinning disk [48, 49, 50] or a high‐speed rotating cylindrical mask can further break through existing modulation rate limitations, while the incorporation of specialized optical materials will simultaneously extend the response capability of the imaging system across a broad spectral range. Moreover, a polygonal mirror to efficiently combine the DMD with laser scanning hardware can achieve pattern projection rates of up to MHz levels [51]. These technological advancements could enable our WLSI to fully leverage its advantages and further promote its applications involving real‐time amplitude and phase imaging of biological samples, defect detection of optical components, wavefront detection in special spectra, etc.

## Conflicts of Interest

The authors declare no conflicts of interest.

## Supporting information




**Supporting File 1**: advs75485‐sup‐0001‐SuppMat.docx.

**Supporting File 2**: advs75485‐sup‐0002‐Video S1.mp4.

**Supporting File 3**: advs75485‐sup‐0003‐Video S2.mp4.

## Data Availability

The data that support the findings of this study are available from the corresponding author upon reasonable request.
