# Supplementary material for: Wavefront Line‐Scan Imaging Via a Single‐Pixel Detector
Source: Adv Sci (Weinh). 2026 Apr 30;13(41):e75485. doi: 10.1002/advs.75485 (PMC13335502; doi:10.1002/advs.75485)
Supplement: Supplementary file 1 — Supporting File 1: advs75485‐sup‐0001‐SuppMat.docx. [file ADVS-13-e75485-s002.docx]

**Supporting Information**

**Supporting Information for “Wavefront line-scan imaging via a single-pixel detector”**

*Nuo Liu,^1^ Aiping Zhai,^1,2^ Tingting Zheng,^1,2^ Wenjing Zhao,^1,2^* Dong Wang^1,2,3^**

^1^ College of Physics and Optoelectronics Engineering, Taiyuan University of Technology, No. 79 West Main Street, Yingze, 030024, PR China

^2^ Shanxi Key Laboratory of Precision Measurement Physics, Taiyuan University of Technology, No. 79 West Main Street, Yingze 030024, PR China

^3^ Key Laboratory of Advanced Transducers and Intelligent Control System, Ministry of Education, and Shanxi Province, Taiyuan University of Technology, No. 79 West

^*^Email: wangdong@tyut.edu.cn.

**Supplementary 1. Mathematical derivation of phase-shifting WLSI**

The modulation basis for spatially sampling the target wavefront can be a random basis or orthogonal bases, such as the Hadamard basis, the Fourier basis, the discrete cosine transform (DCT) basis, etc.

Each sampling pattern ($P_{n}(x)$ $(n\in N^{+})$) has a spatial resolution $1\times N$, and four steps of phase-shifting (denoted asare encoded into the sampling patterns. The *n*th encoding pattern can be obtained, where $S(x)$ represents the signal field and $R(x)=1-S(x)$ is the reference field. Each encoded pattern not only spatially samples the target wavefront but also introduces the four-step phase-shifting. Then, the wavefront of the target object $O_{m}(x)=U_{m}(x)e^{iV_{m}(x)}$ is modulated by the encoded patterns, where $O_{m}(x)$ is the $m\text{th}$ row of the target wavefront. The interference intensity detected by the single-pixel detector can be expressed as

where FFT{} represents the one-dimensional Fourier transform, $x$ represents the spatial domain coordinate, and$w$represents the transform domain coordinate.$w=0$denotes taking the zero-frequency component$S_{m,n}=\int P_{n}(x)\cdot S(x)\cdot O_{m}(x)dx$and. $C_{m, 2}$represents the magnitude of this row’s complex constant$C_{m,1}$, whiledenotes its phase. Thus

The detected intensity values corresponding to the four-step phase-shifting can be,

The corresponding spectral coefficient obtained can be,

The $m\text{th}$ row of the target wavefront can be reconstructed using the second-order correlation (SOC) algorithm,

$$\hat{O}_{m}(x)\approx\frac{1}{N}\sum_{n=1}^{N} Y_{m,n}\cdot P_{n}(x)$$

The complete target wavefront can be expressed as follows,

$$\hat{O}(x)=[\hat{O}_{1}(x),...,\hat{O}_{m}(x),...,\hat{O}_{M}(x)]$$

where *M* denotes the longitudinal resolution of the target wavefront.

**Supplementary 2. Mathematical derivation of off-axis WLSI**

In off-axis interference, a horizontal phase grating is designed to introduce the necessary phase difference, where $i$ is the imaginary unit, and is the phase of the grating. The signal field is represented as $S(x)$, and the reference field is $R(x)=1-S(x)$. The *n*th encoding pattern is obtained by superposing sampling patterns with the phase grating. Then, the wavefront of the target object $O_{m}(x)=U_{m}(x)e^{iV_{m}(x)}$ is modulated by the encoded patterns, where $O_{m}(x)$ is the *m*th row of the target wavefront. The off-axis interference intensity is collected by a lens and detected by a single-pixel detector, which can be expressed as

where$S_{m,n}=\int S(x)\cdot P_{n}(x)\cdot O_{m}(x)dx$and. Since the horizontal phase is known and fixed despite it is related to the coordinate vector $w$, $C_{m,1}$can be considered as this row’s complex constant. Thus, $Y_{m,n}$can be expressed as

$$\begin{matrix} Y_{m,n}=\left|S_{m,n}+C_{m,1}\right|^{2} \\ =\left|S_{m,n}\right|^{2}+\left|C_{m,1}\right|^{2}+S_{m,n}^{*}\cdot(C_{m,1})+S_{m,n}\cdot(C_{m,1})^{*} \\ =I_{S_{m,n}}+I_{C_{m}}+I_{AC_{m,n}} \end{matrix}$$

where,

$I_{S_{m,n}}$and$I_{C_{m}}$represent autocorrelation, $I_{AC_{m,n}}$is the complex conjugate cross-correlation term. The final off-axis hologram can be reconstructed via the SOC algorithm. The $m\text{th}$ row of the final off-axis hologram can be expressed as

$$\begin{matrix} \hat{H}_{m}(x)=\frac{1}{N}\sum_{n=1}^{N} P_{n}(x)\cdot Y_{m,n} \\ =\frac{1}{N}\sum_{n=1}^{N} P_{n}(x)\cdot(I_{S_{m,n}}+I_{C_{m}}+I_{AC_{m,n}}) \\ =\frac{1}{N}\sum_{n=1}^{N} P_{n}(x)\cdot\left(|S_{m,n}|^{2}+|C_{m,1}|^{2}\right)+\frac{1}{N}\sum_{n=1}^{N} P_{n}(x)\cdot\left[S_{m,n}^{*}\cdot(C_{m,1})+S_{m,n}\cdot(C_{m,1})^{*}\right] \\ =\frac{1}{N}\sum_{n=1}^{N} P_{n}(x)\cdot\left(|S_{m,n}|^{2}+|C_{m,1}|^{2}\right)+C_{1}^{*}\cdot\frac{1}{N}\sum_{n=1}^{N} P_{n}(x)\cdot S_{m,n}+C_{m,1}\cdot\frac{1}{N}\sum_{n=1}^{N} P_{n}(x)\cdot S_{m,n}^{*} \\ =\frac{1}{N}\sum_{n=1}^{N} P_{n}(x)\cdot\left(|S_{m,n}|^{2}+|C_{m,1}|^{2}\right)+C_{m,1}^{*}\cdot\frac{1}{N}\sum_{n=1}^{N} P_{n}(x)\cdot\int S(x)\cdot P_{n}(x)\cdot O_{m}(x)dx \\ +C_{m,1}\cdot\frac{1}{N}\sum_{n=1}^{N} P_{n}(x)\cdot\left(\int S(x)\cdot P_{n}(x)\cdot O_{m}(x)dx\right)^{*} \\ =\frac{1}{N}\sum_{n=1}^{N} P_{n}(x)\cdot\left(|S_{m,n}|^{2}+|C_{m,1}|^{2}\right)+C_{m,1}^{*}\cdot\hat{O}_{m}(x)+C_{m,1}\cdot\left[\hat{O}_{m}(x)\right]^{*} \end{matrix}$$

The complete off-axis hologram can be expressed as follows,

$$\hat{H}(x)=[\hat{H}_{1}(x),...,\hat{H}_{m}(x),...,\hat{H}_{M}(x)]$$

where *M* denotes the longitudinal resolution of the target wavefront.

Then, according to the Fourier fringe analysis (FFA), the off-axis interferogram is processed via a Fourier transform to obtain its spectrum. The first-order component of this spectrum is then extracted and translated to the center of the Fourier spectrum. Following this, the target wavefront is reconstructed by conducting the inverse Fourier transform using the extracted Fourier spectrum.

**Supplementary 3.** **Simulations of WLSI using different modulation bases**

Simulations verified the feasibility and versatility of the proposed method using orthogonal bases (Hadamard basis, Fourier basis, and discrete cosine transform (DCT) basis) and non-orthogonal bases (random basis). The target wavefront, with a resolution of 256 × 256 pixels, is shown in Fig. S1(a), where the amplitude is restricted in a circle, and the phase is represented by a grayscale ‘house’ image with varying phase depths. The simulation results of phase-shifting WLSI and off-axis WLSI are shown in Figs. S1(b)-S1(i).

The results demonstrate that the proposed WLSI can reconstruct the wavefront for complex grayscale targets using the four different modulation bases, whether it is based on the phase-shifting WLSI or the off-axis WLSI. From the simulations, the imaging result of different modulation bases is slightly different. Hadamard is an ideal coding scheme for DMD modulation due to its binary nature. Furthermore, it’s worth noting that the method described here is not confined to the four modulation bases mentioned above.


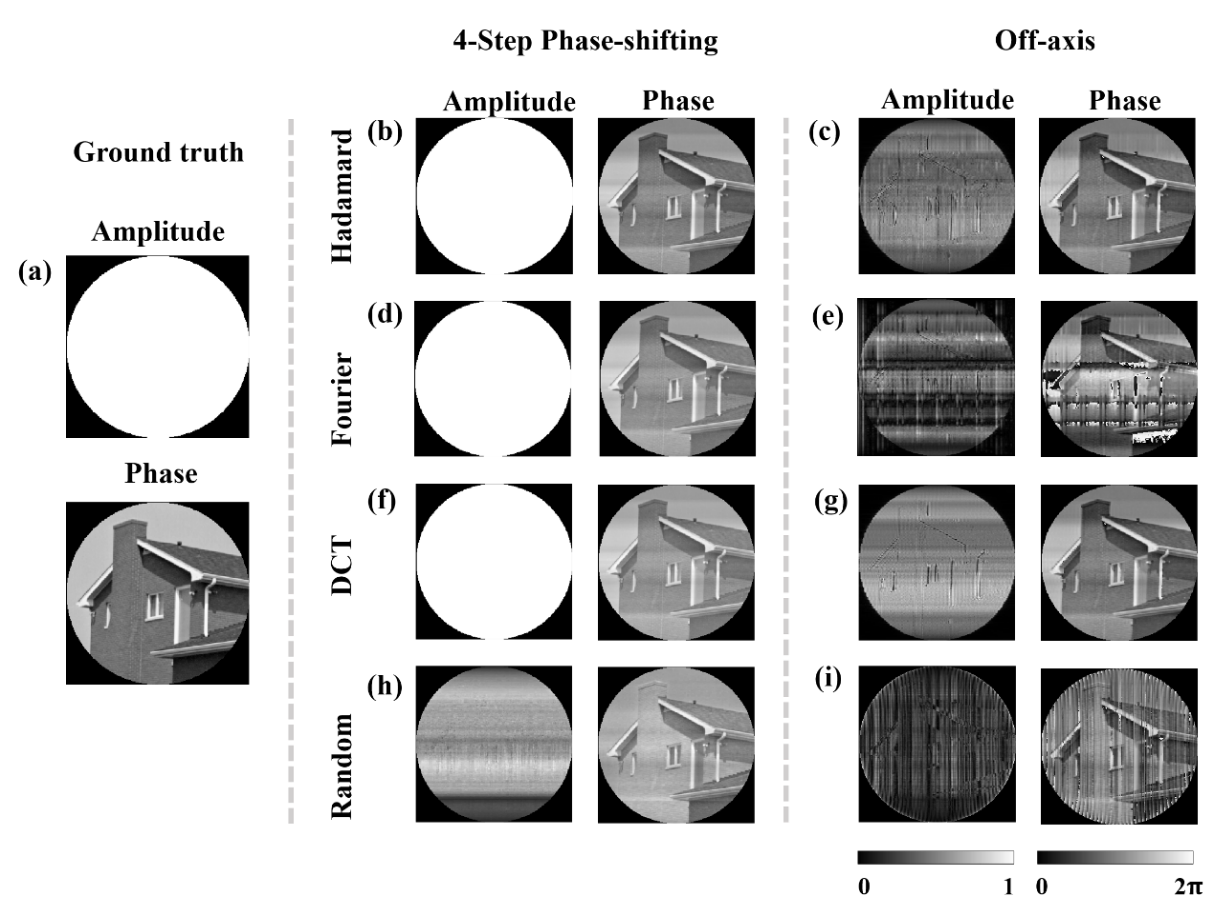


**Figure S1.** Simulation results of WLSI using different modulation bases. (a) Amplitude and phase of the target wavefront. (b-c), (d-e), (f-g) and (h-i) are the simulation results using the Hadamard basis, Fourier basis, DCT basis and random basis, respectively.

**Supplementary 4. Simulations and experiments of WLSI using different reference strategies**

In addition to the peripheral reference strategy (up and down), the proposed WLSI can also use other reference strategies, such as the checkerboard reference strategy, self-referenced strategy, etc. Figures S2(a)-S2(e) schematically illustrate the signal part and the reference part for these different reference strategies. Subsequently, comparisons of the simulation and experimental results with different reference strategies for phase-shifting WLSI are shown in Figs. S2(f)-S2(k).


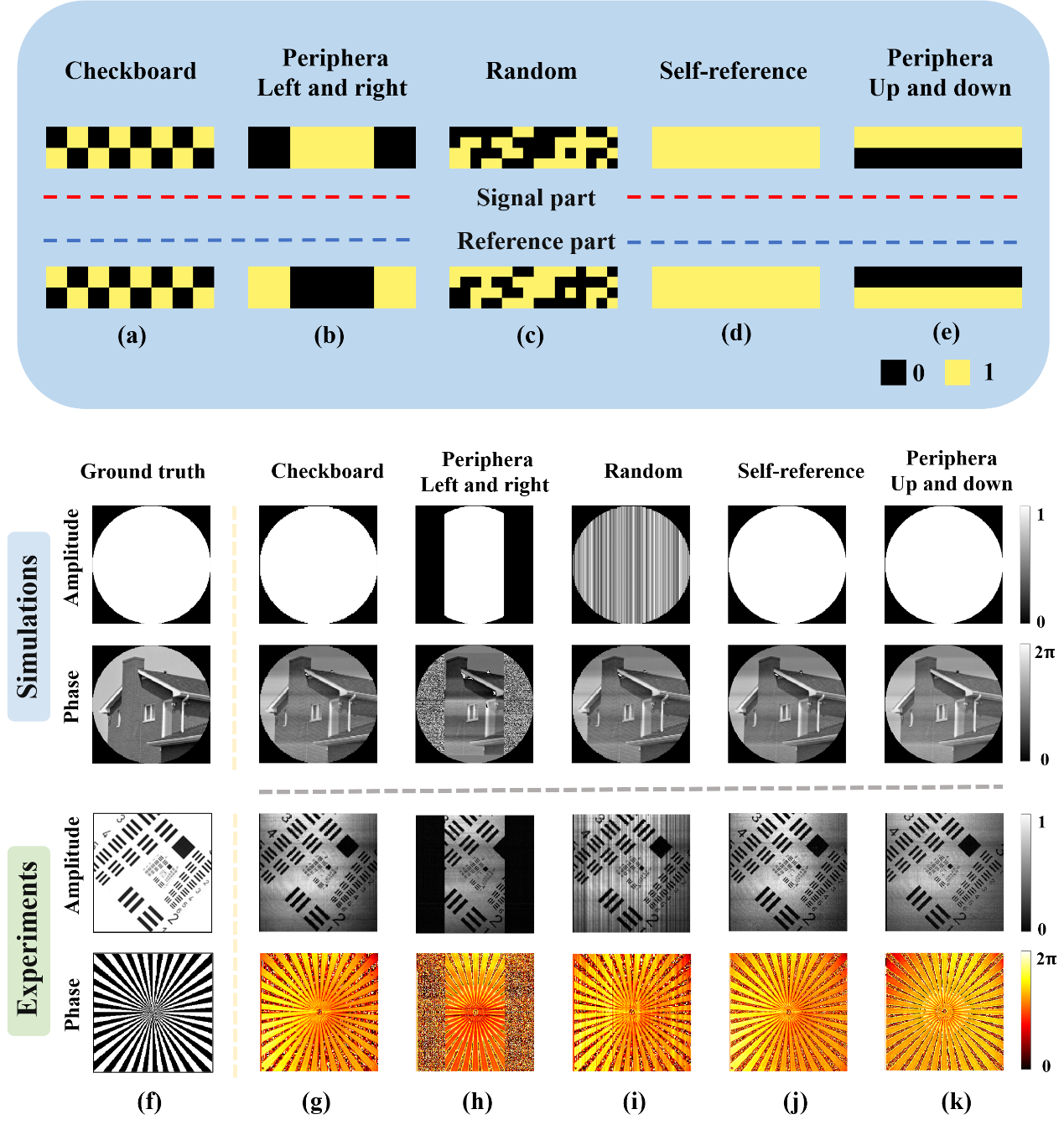


**Figure S2.** Simulations and experiments of WLSI using different reference strategies (a-e) Schematic diagrams of the signal part and the reference part under different reference strategies. (f) shows the ground truths for simulations and experiments. (g-k) show the results of simulations and experiments of WLSI using different reference strategies, respectively.

As can be observed in Fig. S2(g), the checkerboard reference strategy preserves the full field of view (FOV) but sacrifices half of the imaging resolution because half of the DMD micromirrors are allocated for reference. In contrast, the peripheral reference strategy (left and right), as shown in Fig. S2(h), yields a higher imaging resolution while resulting in a smaller FOV than the checkerboard method. This is because it uses the left and right peripheral micromirrors of the DMD as the reference part. As shown in Fig. S2(i), when using the random reference strategy, the image quality is seriously degraded due to the uneven random distribution between the reference part and the signal part. The self-referenced strategy has a good performance when using the binary grating method for phase modulation, but the reconstruction quality is severely degraded when the Lee method is used. The peripheral reference-based (up and down) strategy, as shown in Fig. S2(k), can balance imaging resolution and imaging FOV, and is suitable for different phase modulation methods. Based on the consideration of imaging FOV, imaging resolution, and imaging effect under different phase modulation strategies, this paper selects the peripheral reference strategy (up and down) to demonstrate the proposed method.

**Supplementary 5.** **Simulations and experiments of WLSI using the different complex amplitude modulation methods.**

Here, different phase modulation methods are used for different interference strategies. Figure S3 shows the simulation and experiments of phase modulation methods under two interference strategies. Figures S3(a) and S3(b) are the ground truth of the targets in simulations and experiments. Figures S3(c)-S3(d) and S3(e)-S3(f) show the simulation and experiments using binary grating modulation and Lee's method modulation under phase-shifting interference, respectively. Figures S3(g) and S3(h) show simulation and experiments using the Lee method under off-axis phase-shift interference.

As shown in Fig. S3, in phase-shifting interference, the binary grating naturally aligns with the binary modulation of a DMD, providing the best imaging quality. Therefore, we use a binary grating for the final phase modulation. The off-axis interference introduces the necessary tilt angle by encoding a complex phase grating, where phase modulation using a binary grating is not suitable. Therefore, we use the Lee method for phase modulation, which is binarized to match the DMD modulation.

**
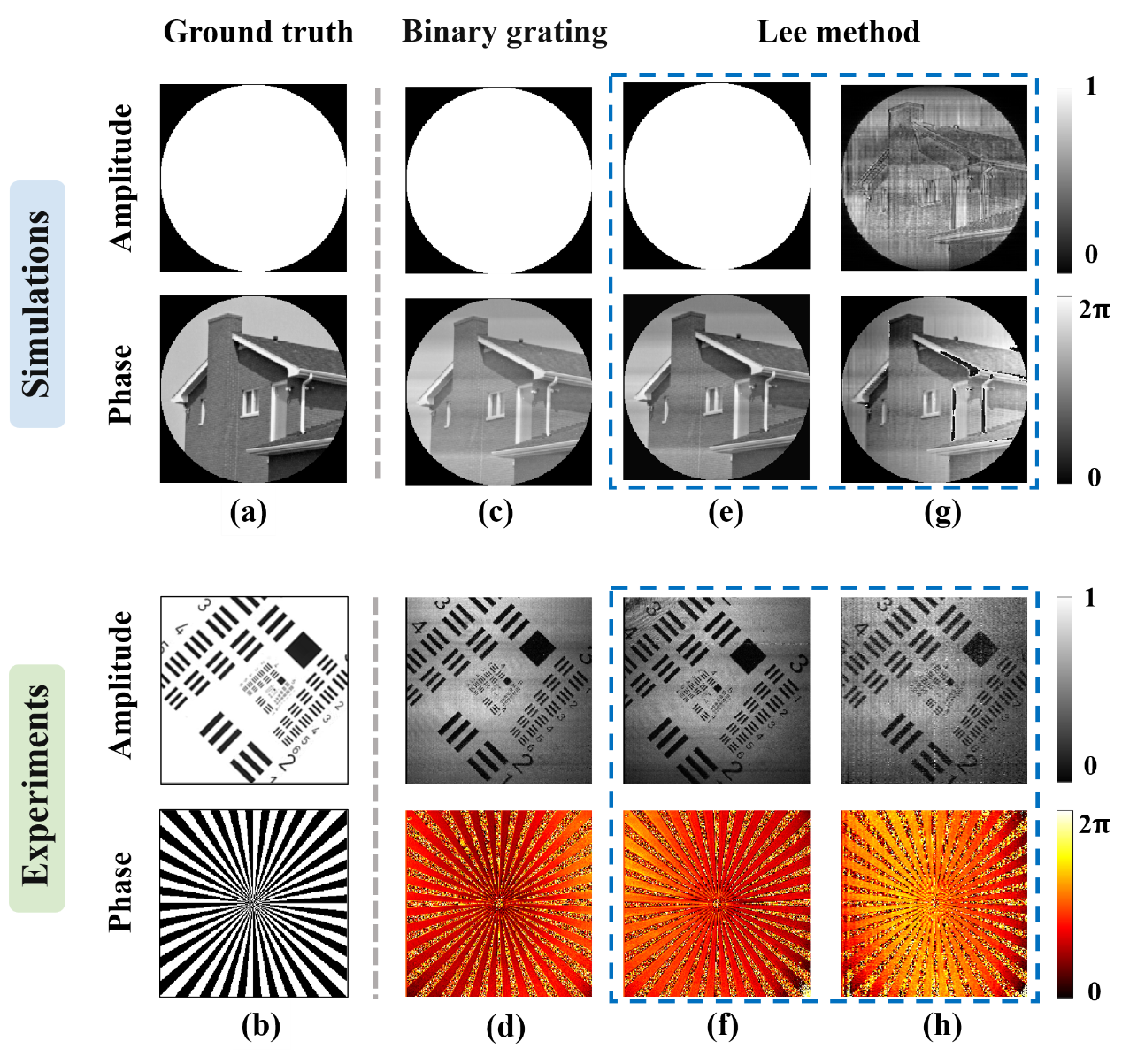
**

**Figure S3.** Simulations and experiments of WLSI using different complex amplitude modulation methods. (a) and (b) are the ground truths for simulations and experiments. (c-d) and (e-f) show the simulations and experiments using binary grating modulation and Lee's method modulation under phase-shifting interference, respectively. (g)-(h) show the simulations and experiments using the Lee method with off-axis interference.

**Supplementary 6.** **Simulation and experiments of WLSI at different sampling ratios.**

To further improve the imaging speed of WLSI, a down-sampling strategy was introduced. The simulations at different sampling rates are presented in Fig. S4(c) , and the corresponding Structural Similarity Index (SSIM) and Peak Signal-to-Noise Ratio (PSNR) values are shown in Fig. S5.

The results show that reducing the sampling rate leads to a gradual decline in reconstruction quality. Nevertheless, the deterioration remains moderate within a certain range. For example, at a sampling rate of 25%, the reconstructed images still preserve clear structural contours. The SSIM values of the amplitude and phase remain as high as 0.9733 and 0.8615, respectively, and the PSNR stays around 35 dB, indicating that acceptable reconstruction quality is maintained.

To further verify the effectiveness of this strategy in practice, we carried out the corresponding imaging experiments, and the results are shown in Fig. S4(d). Consistent with the simulation results, satisfactory reconstruction quality can still be achieved in experiments at 25% sampling rate. Meanwhile, this strategy also brings remarkable improvement in imaging speed. For a resolution of 256 × 256 pixels, the line reconstruction speed is reduced from 2.8 ms per row at full sampling to 0.7 ms per row, corresponding to a fourfold improvement in imaging speed.

These results indicate that a moderate down-sampling level, such as 25%, can significantly accelerate imaging while maintaining satisfactory reconstruction quality, providing a practical balance between efficiency and performance.

**
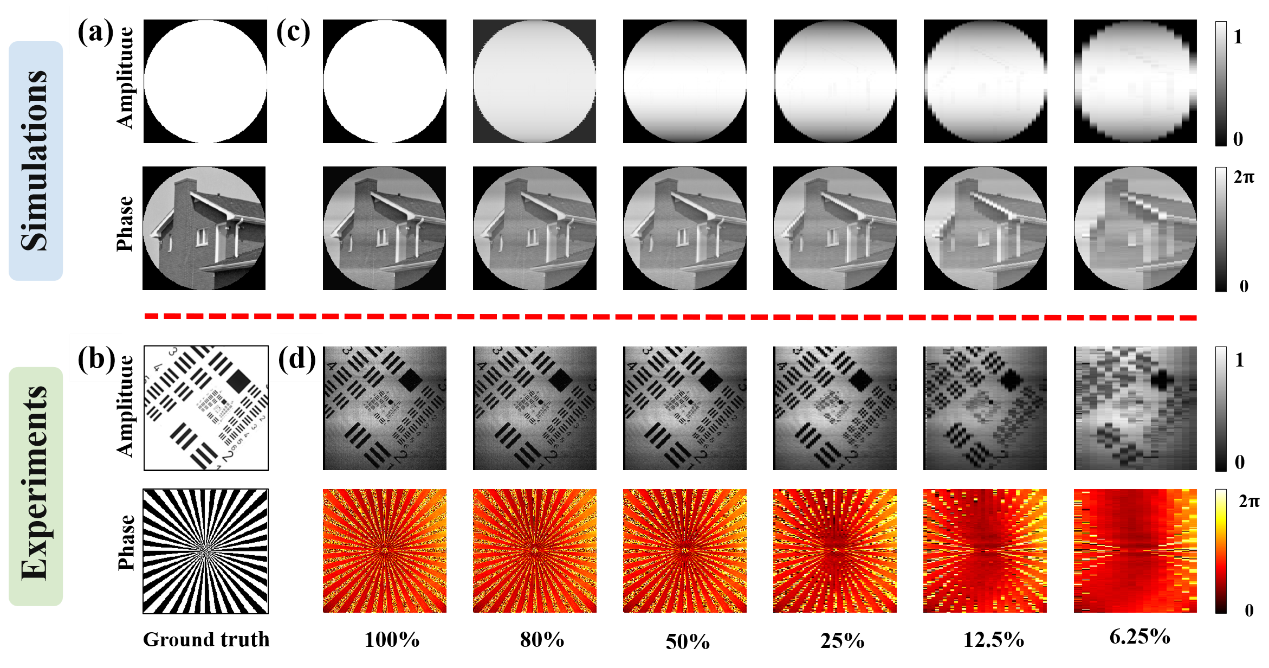
**

**Figure S4.** The simulations and experiments of WLSI at different sampling ratios. (a) and (b) are the ground truths for simulations and experiments. (c) and (d) are the simulations and experiments at different sampling ratios, respectively.

**
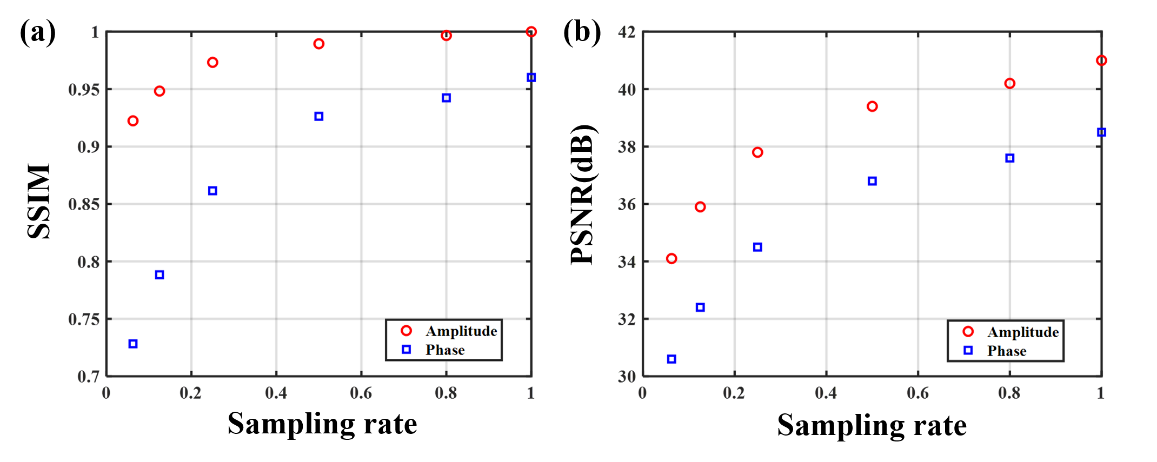
**

**Figure S5.** Reconstruction performance at different sampling rates. (a) SSIM of amplitude and phase reconstruction. (b) PSNR of amplitude and phase reconstruction.

**Supplementary 7.** **Experimental setup.**

The experimental setup of WLSI is shown in Fig. S6. A 532 nm continuous-wave laser (LR-GSP-532) is used as the coherent illumination source, whose beam is amplified by an expander to match the DMD (Vialux V-6501) modulation area. The transmitted beam through the sample is scaled-conjugated to the DMD plane via a 4-f optical system comprising lenses L1 and L2 (focal length: 50 mm). A DMD scrolls the loaded Line-scan patterns (LPs) for wavefront modulation. The modulated beams reflected from DMD are then split to facilitate dual detection schemes, with one arm for phase-shifting detection [Fig. S6(a)] and another arm for off-axis detection [Fig. 6(b)]. The measured wavefront passes through the converging lens (L3/L4) with a focal length of 150 mm. Then it is directed to a photodetector (PD1/PD2, KG-PR-200K-A-FS) with a 20 μm slit (S1/S2), which separates the center of the first-order diffracted component. The output is sampled by a data acquisition device (NI DAQ-USB-6216) and transmitted to a computer for image reconstruction.

**
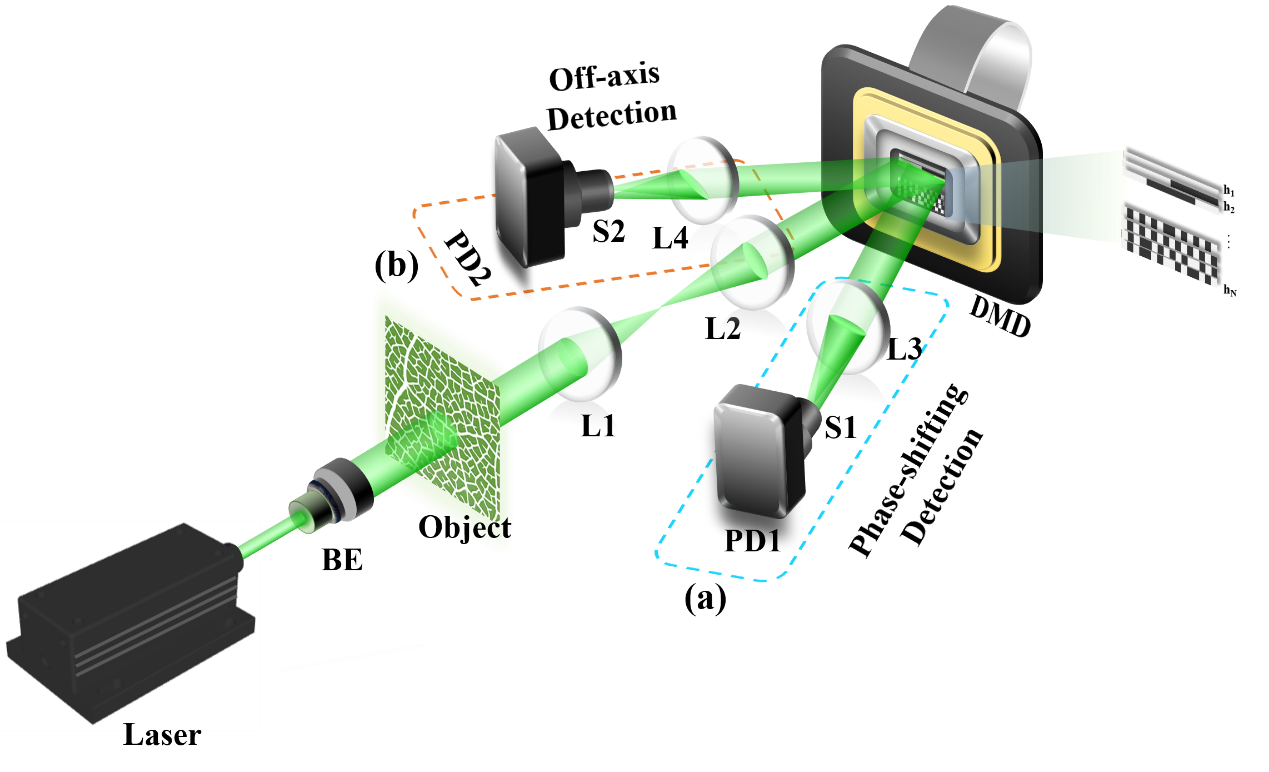
**

**Figure S6.** Experimental setup. (a) One arm for phase-shifting detection. (b) Another arm for off-axis detection. L, lenses. S, slits. PD, photodetector.

**Supplementary 8.** **Evaluation of the minimum resolvable phase petal radius.**

In the Siemens star phase reconstruction experiment, the minimum analyzable phase petal radius *R* is used as a quantitative metric to characterize the phase resolving capability of the proposed system at high spatial frequencies. The determination procedure is described as follows.


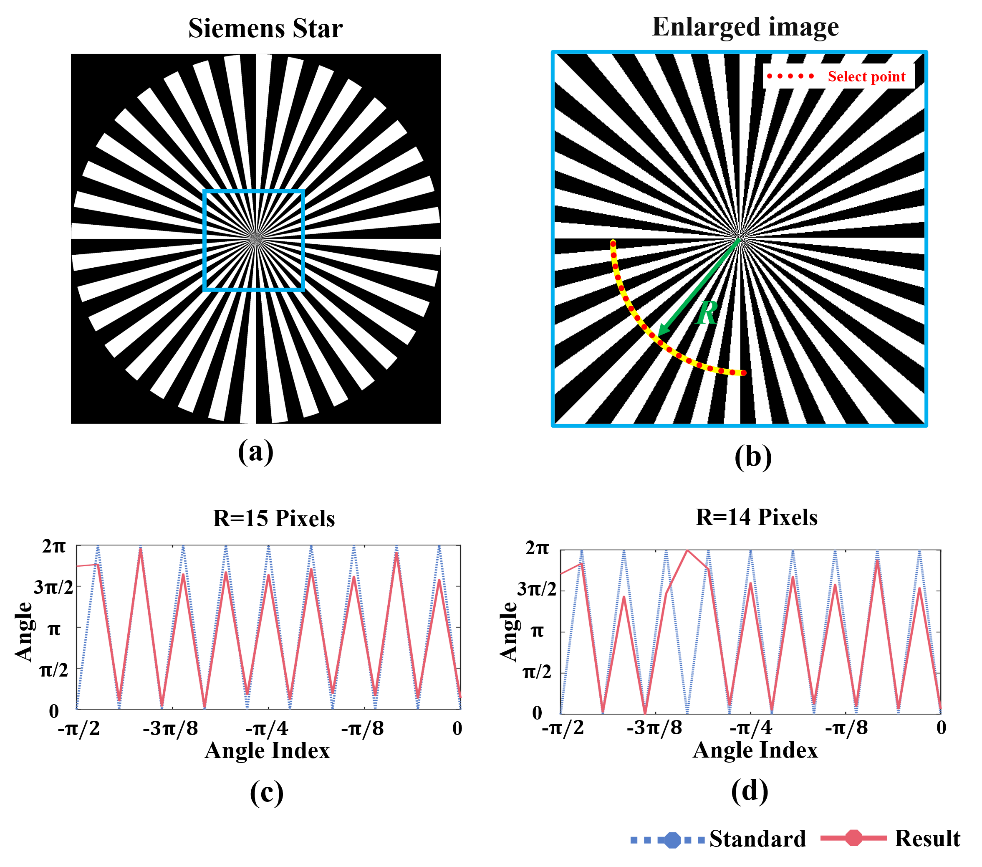


**Figure S7.** Evaluation of the minimum resolvable phase petal radius. (a) Siemens star phase test pattern. (b) Enlarged central region and circular sampling trajectory. (c) Angular phase profile at sampling radius *R*=15 imaging pixels (d) Angular phase profile at sampling radius *R*=14 imaging pixels.

First, based on the known phase distribution of the Siemens star (Fig. S7(a)), a coordinate system is established with the image center as the origin. Figure S7(b) shows an enlarged image of the central region of Fig. S7(a), illustrating the circular sampling trajectory and the angular sampling strategy. Circular trajectories with different radii are selected, and several sampling points (19 points in our implementation) are uniformly distributed along each circumference. The phase values at these locations are extracted from both the theoretical phase distribution and the experimentally reconstructed phase map. A one-dimensional angular phase profile is then constructed by mapping the angular positions of the sampling points to their corresponding phase values, and the reconstructed phase distribution is compared with the theoretical one.

To illustrate this criterion, Fig. 2(f) in the manuscript is taken as an example. When the circular sampling radius is 15 imaging pixels, the reconstructed phase result (Fig. S7(c)) still preserves a periodic variation consistent with the theoretical distribution. When the radius is further reduced to 14 imaging pixels (Fig. S7(d)), the phase profile begins to show obvious distortion, indicating that adjacent phase petals can no longer be reliably resolved. Therefore, 15 imaging pixels are determined as the minimum analyzable phase petal radius of the system.

This corresponds to a physical size of

$$15\times7.6\mu\text{m}\times2=228\mu\text{m}$$

where 7.6 μm is the micromirror size of the DMD (Vialux V-6501), and 2 indicates the number of mirrors combined in the experiment. Therefore, the smallest resolvable phase petal radius of the system is approximately 228 μm.

**Supplementary 9. Wavefront reconstruction simulations using WLSI without and with stitching error compensation.**


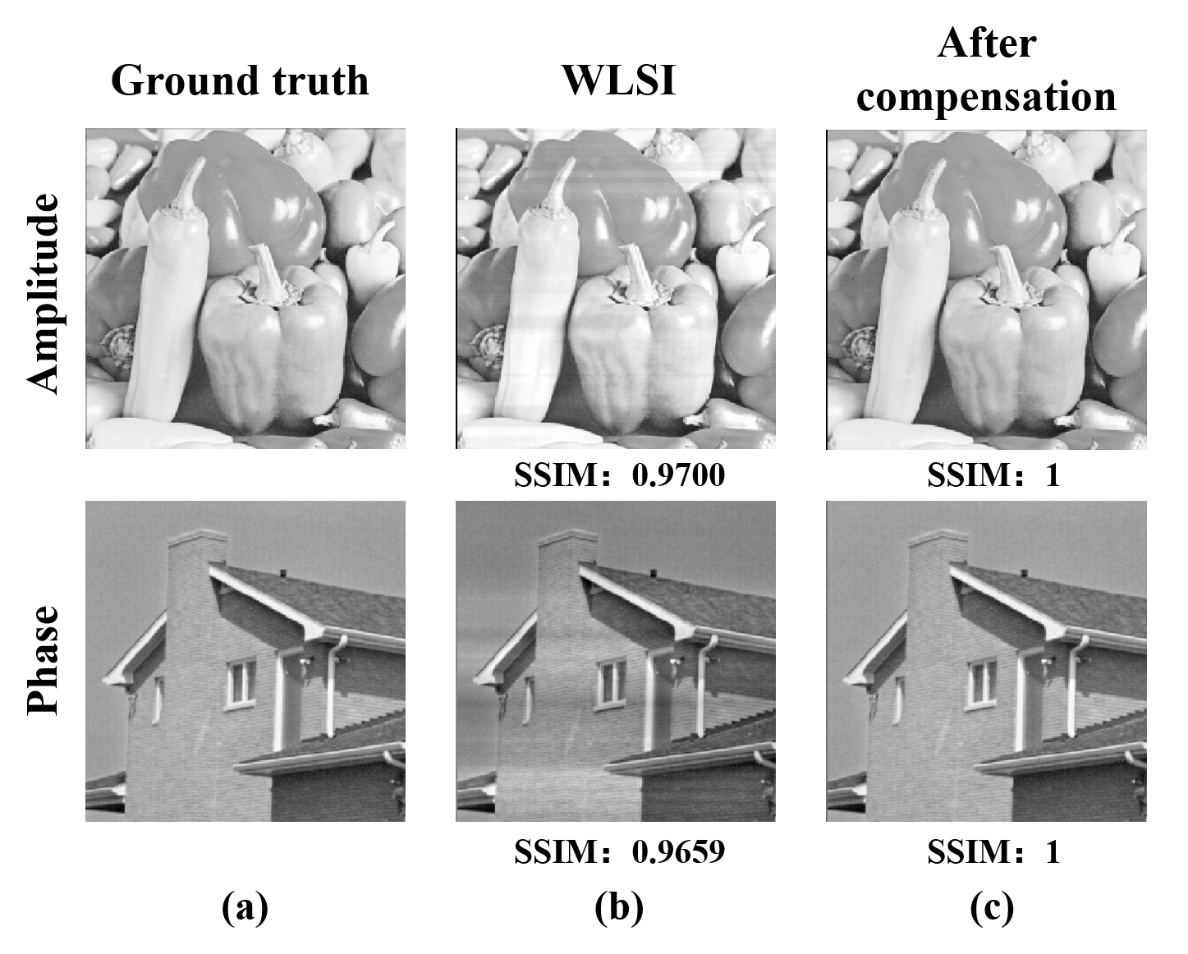


**Figure S8.** Simulation results of WLSI reconstruction for the target at an imaging resolution of 256×256 pixels. (a) The ground truth. (b) The uncompensated reconstruction. (c) The reconstruction after compensating for the row-dependent complex constant *C_m,1_*.


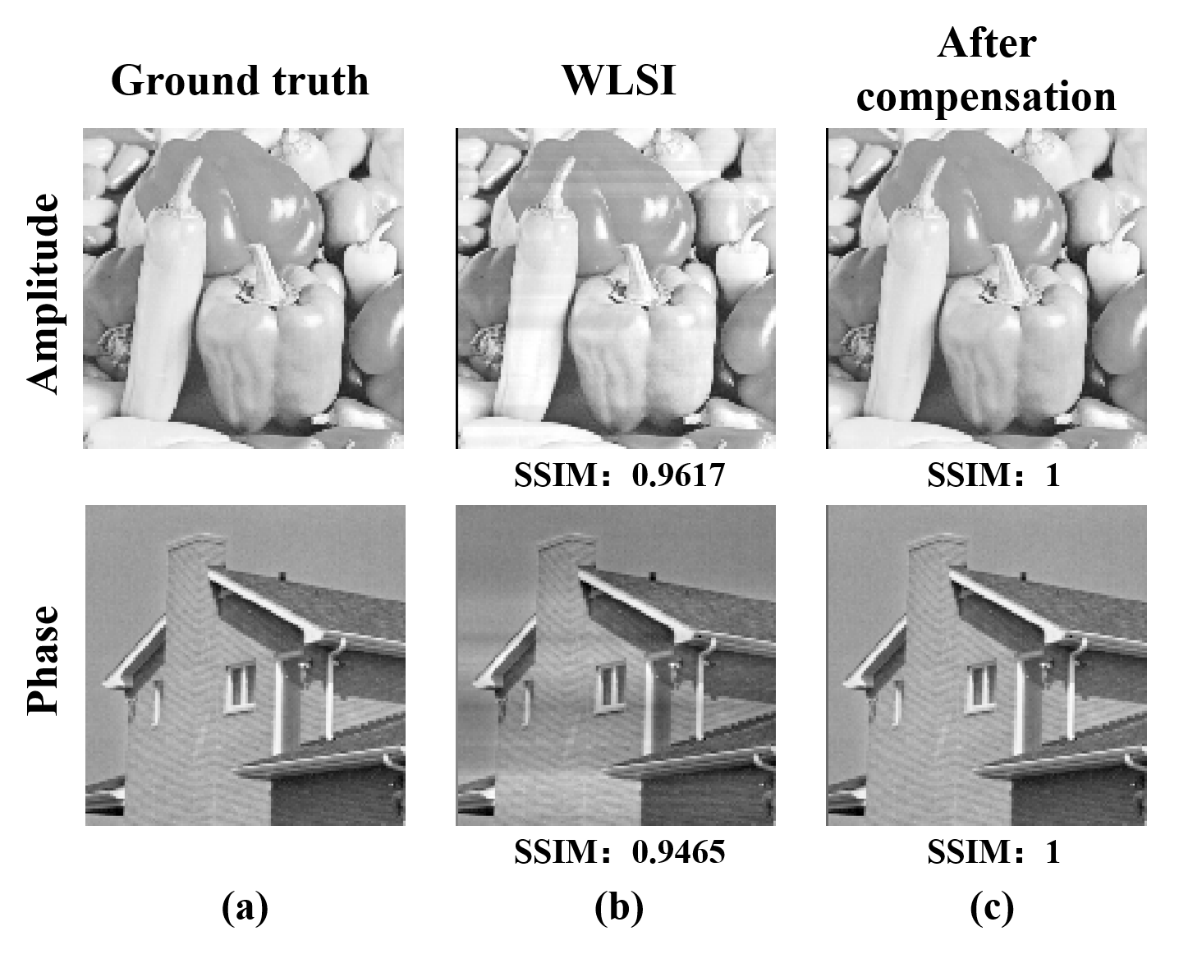


**Figure S9.** Simulation results of WLSI reconstruction for the target at an imaging resolution of 128×128 pixels. (a) The ground truth. (b) The uncompensated reconstruction. (c) The reconstruction after compensating for the row-dependent complex constant *C_m,1_*.
